# Long-term Care Financing: Inserting Politics and Resource Allocation in the Debate: Comment on "Financing Long-term Care: Lessons From Japan"

**DOI:** 10.15171/ijhpm.2019.88

**Published:** 2019-10-15

**Authors:** Cristiano Gori

**Affiliations:** Department of Sociology and Social Research, University of Trento, Trento, Italy.

**Keywords:** Long-term Care, OECD, Financing, Politics, Resource Allocation

## Abstract

The ageing of the countries’ populations, and in particular the growing number of the very old, is increasing the need for long-term care (LTC). Not surprisingly, therefore, the financing of LTC systems has become a crucial topic across the Organisation for Economic Co-operation and Development (OECD). In the last three decades, various financing policies have been carried out in different countries and the related international debate has grown. The latter has so far focused mostly on the different alternatives to collect economic resources to pay for care. The international debate needs now to focus also on other issues, so far less discussed. One is the politics of LTC: the degree and nature of the political interest in LTC, that affects the size and profile of public financing. The other is resource allocation: how different services and benefits are distributed among people with different care needs, that determines if resources made available are optimized. If we do not pay more attention to these issues – inextricably connected to policies aimed to collect funds – our understanding of LTC financing will remain inevitably limited.

The growing number of people living to older and older ages produces increasing pressure on the financing of long-term care (LTC) systems.^1^ In his editorial, Prof. Ikegami discusses several topics concerning LTC financing focusing on Japan.^2^ This commentary enlarges the view to the wider the Organisation for Economic Co-operation and Development (OECD) context.

Since the 1990s, financing LTC has been a key policy issue across OECD. Some countries introduced major reforms to enlarge public responsibility – such as Austria (1993), Germany (1995), Japan (2000), France (2002), Spain (2006), and South Korea (2008) – that, in many cases, have been later modified; mostly – eg, in Austria, Japan, and Spain – those subsequent changes aimed to contain public expenditure.^3^ Elsewhere, reforms to reduce public responsibility were introduced, such as in the Netherlands (2015) and Australia (2014).^4^ In Scandinavia, furthermore, a trend in decreasing support in traditionally comprehensive publicly funded LTC systems has occurred.^5^ In countries such as Britain and the US reforms have been long discussed but not enacted.^6^

Simultaneously, the international debate has considerably grown. The debate has focused on the different alternatives to finance LTC, ie, to collect economic resources to pay for care (social insurance, private insurance, tax-based support and self-based financing, concerning the individual and/or the family), their various designs and the related implications.^7-9^ Although more research is needed, the degree of knowledge concerning different financing options is nowadays undoubtedly significant. Less discussed internationally have been crucial issues that, although inextricably connected to policies aimed to collect funds arise – in logical terms – *before* and *after* their own design. I refer to: (*a* ) before financing: degree and nature of the political interest in LTC, that affects the size and profile of public financing; (*b* ) after financing: resource allocation, ie, how different services and benefits are distributed among people with different care needs, that determines if resources made available are optimized. Even if the space available does not allow an in-depth discussion, this commentary aims to illustrate why that if we do not pay more attention to these issues, our understanding of LTC financing will remain inevitably limited.

## Politics of Long-term Care

Any discussion concerning the resources to collect for LTC across OECD has to confront with four matters of fact. First, in many countries it is necessary to augment the public expenditure in the short-term in order to assure appropriate and equitable answers to people with care needs.^10^ Second, in all countries it will be necessary to increase the public expenditure in the mid and long-term; on average, across OECD, the public expenditure as a percentage of gross domestic product is predicted to raise from 0.8% in 2006 to 2.1% in 2060 in an upside “cost-pressure scenario,” and even in the so-called cost-containment scenario to 1.6%.^11^ Third, the push to enlarge LTC public expenditure is to consider along with similar pressures arising from other spending programmes – related to the ageing population (health,^11^ pensions^12^) and to various national priorities – and the room of manoeuvre allowed by the overall government budget. In a number of countries a competition among different public sectors for more funds – mostly unstated but in fact present - is occurring and it is going to grow.^13^ Fourth, the contribution of LTC private insurance to alleviate the pressure on the public budget is going to be moderate. Private insurance is currently scarcely developed across OECD, and there is consensus in the literature that the capacity of these products to reduce the need for public expenditure will be – in most countries – limited.^7^ Private insurance, in fact, is mostly expected to supplement public expenditure not to reduce the need for the former.

In brief, a substantial increase of LTC public expenditure is necessary but the goal is going to be – to various degrees according to national circumstances – quite challenging. The actual possibility to achieve this goal depends, first and foremost, on political choices. The preliminary condition, in fact, resides in governments’ will to invest in LTC in a scenario marked by several constraints. The topic is the politics of LTC.

The comparative discussion on the politics of LTC has been so far quite limited, although with notable exception.^14,15^ A more in-depth examination of the reasons leading governments to assign political priority to LTC, or not, is needed. This is a next step for the debate: the advanced reflections on the “how” question (how to design financing policies) should be accompanied by an increasing investigation of the “why” question, looking at the causes, factors and circumstances associated with a high or low political priority assigned to LTC. The “why” question concerns policy legacy, cultural norms, institutional frame, social and economic context, role of political and social actors, broad political context and so on. A glance at both the various national trajectories in LTC financing occurred since the nineties across OECD – mentioned above – and the different reactions to recent times of austerity^16-18^ highlights the importance of the political variable. In brief: an extensive knowledge on how to collect public resources should be matched by an equally extensive understanding of the reasons why politicians aim (or not) to devote more resources to LTC.

## Resource Allocation

Prof. Ikegami suggests that, in publicly funded LTC systems, “key issue is not necessarily costs, but resource allocation.”^2^ The latter can be examined from – at least – two different perspectives: the mix of public services and benefits provided (cash benefits, community services in kind, residential care) and the balance between coverage and intensity. By coverage it is meant the percentage of target population receiving public care and by intensity the amount of care per user provided (measured, for example, by periodical unit costs or – in in-kind community services – by number of periodical visits).

Patterns of resource allocation have been quite neglected so far in the international literature on LTC.^19^ The main reason is well known: the scarce availability of comparable data. Nevertheless, such a difficulty should not prevent from paying more attention to the topic in the future.^20^ How to best allocate public resources is always a main challenge and it is going to be even more important in the years to come, due to the pressure produced by the increasing older population. Here it is the key point: an in-depth debate on how to collect public resources is going to be inevitably partial if not accompanied by an equally in-depth debate on how to allocate these resources at the best.

For example, Prof. Ikegami refers to the unexpected findings of a comparison of per capita LTC public expenditure for the population 65 and over in seven countries.^21^ In order to understand these findings – he argues – it is necessary to focus on the different mixes of care services and benefits provided. Another example consists in a comparison of some OECD countries (England, Germany, Italy, Japan, Sweden, and the United States), focused on older people too, we conducted to investigate whether coverage or intensity have been prioritized since the beginning of the century. Some consistent trends emerged: in all countries coverage has been prioritized over intensity in care provided in the community whereas the opposite occurred in residential care (both publicly funded cash benefits and services in kind were taken into account).^22^ In the latter, there was a trend to reduce the share of older people who received LTC services and to focus on the most serious cases, increasing the public expenditure per user. In community care, the predominant trend consisted in an increase of coverage, accompanied either by a reduction in intensity or by an increase in intensity of a lower percentage than coverage. These findings are confirmed by other studies.^23,24^ The trends depicted highlight several tensions between coverage and intensity – connected to the mix of care inputs provided – that policy-makers will have to address in the near future in order to optimize resource allocation.

The international debate on LTC financing has, so far, focused on the question “how the resources to pay for LTC can be collected?” This commentary has argued the need to broaden the view, inserting politics and resource allocation into the picture. The question mentioned above would, therefore, part of a broader frame, structured into three analytical questions, that are logically consecutive: (1) “why politicians aim (or not) to devote more resources to LTC?” (2) “how the resources can be collected?” (3) “how their allocation can be optimized?” The goal consists in providing a comprehensive view of the financing issue, in its different and interrelated elements.

## Ethical issues

Not applicable.

## Competing interests

The author certifies that they have no afﬁliations with or involvement in any organization or entity with any ﬁnancial interest (such as honoraria; educational grants; participation in speakers’ bureaus; membership, employment, consultancies, stock ownership, or other equity interest; and expert testimony or patent-licensing arrangements), or non-ﬁnancial interest (such as personal or professional relationships, afﬁliations, knowledge or beliefs) in the subject matter or materials discussed in this manuscript.

## Author’s contribution

CG is the single author of the paper.
